# The Power of Phytochemicals Combination in Cancer Chemoprevention

**DOI:** 10.7150/jca.34374

**Published:** 2020-05-18

**Authors:** Balsam Rizeq, Ishita Gupta, Josephine Ilesanmi, Mohammed AlSafran, MD Mizanur Rahman, Allal Ouhtit

**Affiliations:** 1 Department of Biological and Environmental Sciences, College of Arts and Sciences, Qatar University, Doha, Qatar; 2 Biomedical Research Center, Qatar University, Doha, Qatar; 3 College of Medicine, Qatar University, Doha, Qatar

**Keywords:** Complementary alternative medicine, Cancer, Phytochemical combination, Genomics, Review

## Abstract

Conventional therapies for cancer treatment have posed many challenges, including toxicity, multidrug resistance and economic expenses. In contrast, complementary alternative medicine (CAM), employing phytochemicals have recently received increased attention owing to their capability to modulate a myriad of molecular mechanisms with a less toxic effect. Increasing evidence from preclinical and clinical studies suggest that phytochemicals can favorably modulate several signaling pathways involved in cancer development and progression. Combinations of phytochemicals promote cell death, inhibit cell proliferation and invasion, sensitize cancerous cells, and boost the immune system, thus making them striking alternatives in cancer therapy. We previously investigated the effect of six phytochemicals (Indol-3-Carbinol, Resveratrol, C-phycocyanin, Isoflavone, Curcumin and Quercetin), at their bioavailable levels on breast cancer cell lines and were compared to primary cell lines over a period of 6 days. This study showed the compounds had a synergestic effect in inhibiting cell proliferation, reducing cellular migration and invasion, inducing both cell cycle arrest and apoptosis. Despite the vast number of basic science and preclinical cancer studies involving phytochemicals, the number of CAM clinical trials in cancer treatment still remains nascent. In this review, we summarize findings from preclinical and clinical studies, including our work involving use of phytochemicals, individually as well as in combination and further discuss the potential of these phytochemicals to pave way to integrate CAM in primary health care.

## Introduction

Health challenges pose major burdens on population economic standards, life expectancy and mortality rate. Cancer is a major contributor to these global pressures with epidemiological evidence presenting a record of more than 14 million new cases and a mortality rate of approximately 8 million every year 1. The rise in mortality rates is associated with increase in tumor recurrence due to cells becoming resistant to chemo- or radio-therapy 2. Therefore, it is critical to develop an effective alternative strategy to manage and treat cancer is imminent.

Some bioactive molecules have been isolated, characterized and identified as phytochemicals. These phytochemicals are classified based on their chemical composition and structure to carotenoids, organosulfur compounds, and phenolics. Accumulating evidence shows that many of these phytochemicals can target a wide variety of signalling molecules implicated in cellular growth, proliferation, differentiation, and death in a series of cell types 3-6. With an increase in cancer incidence and high mortality rates, a profound understanding of dietary combinations as potential chemo-preventive agents is undoubtedly an attractive strategy to combat cancer and promote global health.

In this review, we will focus on several bioactive phytochemicals as well as their target effectors in cancer.

## Conventional Medicine and Treatment of Cancer

The variations in treatment response suggest intrinsic or acquired therapeutic resistance exists in a subset of cancer patients. This frequently leads to treatment failures, disease progression, and often mortality. Cancer can be treated with both invasive and non-invasive treatment modalities such as surgery, chemotherapy, radiation therapy, as well as other therapeutic modalities including gene therapy, immunotherapy, hormone therapy, photo-dynamic therapy, targeted therapy, palliative care, and a combination of these (e.g. radiosurgery) 7. While, chemotherapy and radiotherapy remain the major standard care for cancer patients as it helps to shrink tumor size and kill cancer cells at the primary sites or metastasizing sites 8, the response to treatment varies substantially in different types of cancer, or even among patients with the same type of cancer. Among the conventional procedures, radiation therapy or radiotherapy is one of the most common treatments for cancer 9. Traditional radiotherapy uses high energy electromagnetic waves, (e.g. gamma rays and X-rays) to destroy and kill tumor cells by damaging their DNA 10. Furthermore, radio-immunotherapy studies in rodent cancer models using radiolabeled derivatives of J591 with α-emitters elicits anti-proliferative potential with minimal damage to the surrounding normal cells 11, 12.

An ideal chemotherapeutic anticancer agent would selectively kill tumor cells but not normal cells. However, most conventional anticancer drugs display non-specificity, causing many undesired toxic effects and patient discomfort 13. In fact, because of the ineffectiveness of current drugs, previous studies have proposed a model where an understanding of the mechanisms underlying tumor-host interactions might lead to the discovery of new drugs that can overcome the resistance issues 14. In addition to pure chemotherapy, specific chemical agents (radio-sensitizers), can generally enhance the cell's response to ionizing radiation as well as promote both direct and indirect effects of radiation 15. Consequently, the development of agents that are capable of selective accumulation in solid tumors represents an exciting new field and forms a key goal of modern anticancer research.

Many targeted therapies, including monoclonal antibodies and pro-inflammatory cytokines, have been developed to target key pathways in tumorigenesis. For instance, interleukin-2 (IL-2) plays an essential role in the immune response by stimulating proliferation of T-cells as well as inducing the generation of cytotoxic T lymphocytes (CTLs) and natural killer (NK) cells; which in turn attack tumor cells. However, despite all these immunity-boosting effects, administration of IL2 in therapeutically active doses is still hampered by excessive toxicities 16. Moreover, as an adjuvant cancer current treatment modality is based on the cytotoxic properties of localized high temperatures (43^o^C) or hyperthermia that is achieved within cancer cells and tissues, the renaissance of this method is mainly based on the modern electromagnetic heating techniques and is usually achieved by using microwaves to heat the tumor 17, 18.

## Shortcomings of conventional therapy

Despite its effectiveness, conventional chemotherapy has many side effects. For instance, cutaneous hypersensitivity reactions are observed in patients receiving treatments based on platinum alkylating agents, topoisomerase and mitotic inhibitors; Hyperpigmentation is prevalent with the use of alkylating agents such as ifosfamide, cyclophosphamide, and thiotepa. Hand and foot syndrome is prevalent in liposomal doxorubicin, daunorubicine and 5'-fluorouracil 19.

The development of biopharmaceuticals as an alternative hybrid therapy also poses limitations. Biologicals are complex anticancer macromolecules such as monoclonal antibodies, antibody fragments and anti-body-drug conjugates. These therapies have clinically displayed low efficacy and weak ability to penetrate solid tumors. Other therapies such as drug delivery systems (DDS) are designed with conventional active drugs molecules attached to biological carriers such as liposomes, nanoparticles or biodegradable polymers 20. It has been reported that molecular target therapies like DDS are linked with ophthalmologic toxicities ranging from blurred vision, conjunctivitis, keratitis, and optical neuritis 21.

One of the major disadvantages of conventional therapies is the recurrence, as not all cancer stem cells are eradicated from the body. In addition, the development of multi-drug resistance (MDR) is a significant clinical challenge. Also, in many countries, social and economic burdens incurred through treatment costs remain among the many hurdles to be overcome for a global reduction in cancer incidence and mortality. Therefore, there is a pressing need to identify novel approaches and therapies that may offer relatively cost-effective regimens with less undesired side effects 22. Many of these complementary and alternative therapies either chemo-preventive or chemotherapeutic are largely inspired by nature, particularly phytochemicals from plants.

## Complementary and Alternative Medicine (CAM) and Role in Cancer Treatment

Complementary and alternative medicine (CAM) involves approaches and compounds that are not classically considered as conventional therapies 23. Indeed, CAM represents the use of naturally occurring or synthetic derivatives of dietary phytochemicals for the treatment and prevention of cancer. CAM encompasses a wide range of therapeutic approaches, including herbal medicines, homoeopathic remedies, and essential oil and dietary supplements. Epidemiological and preclinical studies have identified that both nutritional and behavioural factors may significantly affect the prevalence of some cancers. This has led to increased interest in dietary phytochemicals 24. Contextually, nearly half of cancers diagnosed in the Western world are breast cancers 25. Importantly, this type of cancer is preventable through health diets rich in plant-based products 26, 27. Many dietary phytochemicals are naturally occurring bioactive chemicals that can be efficacious in cancer chemoprevention either independently or in combinations 28.

Most phytochemicals are required in high doses to be effective; these doses are often not achievable through di*et al*one. In general, all types of cancer cells exhibit aberrant gene expression due to mutations in the epigenome because of epigenetic modifications involved in cell proliferation, differentiation and survival 29. Hence, the epigenetic diet was introduced as a chemo-preventive approach related to delineating the efficacy of dietary bioactive compounds and their effect on modulating the epigenome 29.

Nutrigenomics, another promising cancer chemo-preventive strategy, involves the modulation of gene expression in response to dietary compounds. Therefore, a holistic and intuitive approach for chemo-preventive therapy will make use of these bioactive dietary compounds to regulate epigenomic changes and prevention 30. Specifically, realistic approaches focus more on the combinational efficacy of these promising dietary phytochemicals.

## Chemopreventive dietary phytochemicals

Chemopreventive dietary phytochemicals are naturally occurring bioactives found in fruits, vegetables, plants, and spices. They possess anti-inflammatory, anti-oxidative, anti-proliferative and pro-apoptotic properties, and are particularly known to inhibit the growth of various cancer cells. Indeed, a steady inverse effect of increased vegetable and fruit consumption with reduced incidence of oral cancer has been reported 31, 32. Furthermore, dietary phytochemicals have both chemotherapeutic and chemosensitizing effects 33.

## Potential Therapeutic Strategies for Dietary Phytochemicals

Through drug-interaction, the pharmacological effects of dietary phytochemicals demonstrate a positive interaction promoting potency of the bioactive compound *via* molecular interaction with adjuvant substance (potentiation). They exhibit a combined efficacy which is equivalent to the sum of individual effects (additive), or combined efficacy, which is greater than the sum of individual effects (synergistic), and lastly the combined efficacy which is less than the sum of individual effects 34, 35.

## Potent dietary phytochemical combinations

Several epidemiological and case studies have identified the molecular activities of selected phytochemicals that target oncogenic pathways and exhibit chemotherapeutic and/or chemosensitizing effects on cancer cells (Table 1). One area of particular research interest is the identification of the active compounds present in various herbal and dietary interventions and their analysis for anti-cancer properties. For example, polyphenols from green tea, grape seed/skin, anthocyanin and pigments from many flowers, algae, fruits and vegetables are a few of the compounds that have been tested in cancer 36. Tea, the second most consumed beverage in the world, contains polyphenols shown to possess photo-protective effects from UV induced DNA damages causing oxidative stress, inflammation, changes in cell signalling pathways, and epigenetic alterations. There are three major teas based on fermentation process: black, green and oolong 37. The most popular tea polyphenols are the green tea phenols with Epigallocatchin-3-gallate (EGCG), the most abundant (50-88%) component present in green tea 37. A mice model study found that EGCG promotes the repair of UV-induced cyclobutane pyrimidine dimers (CTDs), a chemical directly responsible for DNA damage 38. EGCG also prevents inflammation by reducing cyclooxygenase (COX-2) enzyme, a limiting enzyme for a cascade of carcinogenic pathways promoting skin cancer 38. Recent evidence also indicates that EGCG favorably induced epigenetic changes by modifying miRNA expression in prostate cancer, leading to inhibition of prostate carcinogenesis 39; indicating EGCG's potent antioxidant capacity 40. Recently, EGCG along with curcumin (discussed below) showed potent inhibitory effects against colorectal carcinoma 41. Interestingly, along with its long-term safety, as well as its very negligible side-effects, EGCG makes an attractive bioactive in cancer prevention and treatment 42.

A common property of these majority compounds is their anti-oxidant/free radical eliminating ability. However, few of them infuse high free radical formation to result in the killing/elimination of cancer cells. While several research has analyzed effects of individual compounds derived from grape seeds/skin, tea polyphenols, etc., few research studies determined the combined effects of these compounds when used in synergistic, additive or antagonistic combinations. Most studies have evaluated the effects of individual compounds on a variety of cancer cells *in vitro*. A main draw-back of these studies is the concentration of compounds used typically exceeds their '***bioavailable***' concentration: the serum levels achieved by oral intake of extracts (as practiced in CAM) are much less than used in *in vitro* studies.

### Curcumin

Curcumin, the derivative of turmeric is extracted from the roots of the *Curcuma Longa* plant. Howells *et al* 43 determined several bioactivities of this compound. It downregulates cyclin D1, cyclin E, MDM2 and enhances tumor suppressors p21, p27 and p53 44 leading to arrest of cell cycle, inhibition of proliferation, and induction of apoptosis of a number of cancer cell lines 45. Furthermore, Yan *et al.* identified several putative novel molecular targets of curcumin using gene expression profiling 46. Bioavailability of curcumin seems to be low and plasma levels in nanomolar to micromolar range have been detected after oral administration of this compound 43, 47. The general consensus is that *in vitro* studies with curcumin in the 10 μM range or below might have human physiological relevance. Its role as a chemopreventive agent may lie primarily within the gastrointestinal tract where its concentration is not dependent upon systemic absorption. In spite of low bioavailability, several animal studies have demonstrated the anticancer activity of curcumin in cervical 48, breast 49, prostate 50, liver 51, and lung 52, 53 cancers. Curcumin has been demonstrated to synergize with different agents such as genestin in breast cancer 54, with epigallocatechin-3-gallate in oral cancer 55, with tumor necrosis factor (TNF)- related apoptosis-inducing ligand (TRAIL) on LNCaP prostate cancer cells 56. Curcumin inhibits tumor cell growth and promotes apoptosis *via* modulation of specific carcinogenic biomarkers such as cyclooxygenase-2 (COX-2), nuclear factor kappa (NF-kB), tumor necrosis factor alpha (TNF-α), cyclin D1 and STAT-3 57, 58. In cervical cancer, treatment of Siha cells with curcumin showed a profound decrease in EMT *via* a Pirin-dependent mechanism 48. On the other hand, in non-small cell lung cancer, curcumin showed regulate EGFR and TLR4/MyD88 pathways in combination to downregulate downstream cell cycle‑ and EMT‑related regulators so as to inhibit cell proliferation and metastasis 53.

Recent evidence shows that curcumin targets stem cells of pancreatic cancer 59 as well as reduces inflammation during acute pancreatitis *via* the mitogen‑activated protein kinase‑signaling pathway 60. It has been shown to synergize with other established chemotherapeutic agents such as Oxaliplatin in colorectal cancer cell lines 43, placitaxil in bladder cancer 61, and gemictbine in an orthotopic model of pancreatic cancer. A combination of curcumin and quercetin (a type of Flavonoid) (400/20 mg) was administered to five patients with familial adenomatous polyposis (FAP) and all 5 patients showed reduced polyp size after a period of 6 months dosing compared to the control group 62. Interestingly, a recent report shows that in combination with metformin, curcumin elicits a potent chemo-preventive effect against oral squamous cell carcinoma through a cancer stem cell-driven mechanism 63. Kong *et al.* showed efficacy of dietary curcumin (used at 2% dose) or a combination of curcumin (1%) and PEITC to inhibit high grade carcinoma and increase apoptosis in a transgenic adenocarcinoma of the mouse prostate model accompanied by downregulating the Akt signaling pathway 64. A number of studies have been reported on the effects of curcumin in ovarian cancer 65. Cell cycle inhibition, apoptosis induction, inhibition of ovarian cancer tumor growth by 50%, and angiogenesis in vivo (at 500 mg/kg dose), have been reported as a result of curcumin treatment 65. Inhibition of NF-KappaB pathway, modulation of Akt and p38 MAPK for induction of G2/M arrest/apoptosis 66, enhancement of Apo2L/TRAIL induced apoptosis 67, modulation of drug metabolizing enzymes 68 were demonstrated. In mice models with HER-2 positive breast cancer, curcumin was found to slow tumor formation 69.

### Resveratrol

Resveratrol, a phytoalexin extracted from grapes, red wine, peanuts and berries with highest concentrations is found in Japanese knotweed (*Polygonum cuspidatum*) is a powerful antioxidant. Resveratrol is sometimes used therapeutically for treating inflammation and bacterial infections. It has Cox-1 inhibitory activity 70, 71, causes G1 arrest 72, induces apoptosis by TRAIL sensitization and down regulates survivin expression 73. It has been shown to possess vasorelaxing, anti-inflammatory, anti-lipidemic, anti-estrogenic, antioxidant, anti-fungal and antibacterial properities 74-76. *In-vitro* investigations demonstrated its anticancer potential in a wide variety of tissues including breast, colon, pancreas, stomach, prostate, head and neck, ovary, liver, lung, and cervix 74. It has a wide variety effects on a number of cancer cell lines including LNCaP (prostate), MCF7, T47D, MDA-MB231 (breast), and melanoma 77.

Several research of resveratrol effects on ovarian, breast and prostate cancers were analyzed 78-83. It was shown to inhibit Hypoxia induced Factor-1 alpha and VEGF expression in ovarian cancer cells and was associated with inactivation of p42/p44 MAPK, p70S6K, and enhanced degradation of HIF-1 alpha protein 81. Glucose metabolism was blocked leading to autophagy 79, 80 in a panel of five ovarian carcinoma cell lines when synergized with cisplatin and doxorubicin 84. Microarray analysis of gene expression profile revealed modulation of 118 genes by more than 2 fold after 50 µM RE treatment of PA-1 ovarian cancer cells 71. One of the most highly up-regulated genes was NADPH quinine oxidoreductase 1, which has been shown to be involved in p53 regulation.

Recent work has shown that resveratrol synergizes with vitamin E analog alpha-TEA and methylseleninic acid to inhibit breast cancer cell growth 78. Recent reviews of this compound emphasize its ability to target multiple cellular pathways involved in metabolic syndrome, aging, cardiovascular diseases and cancers 82, 83. *In-vivo* investigations of Harper *et al* 78 demonstrated that resveratrol fed in the diet (at 625 mg/kg dose) to Transgenic Adenocarcinoma Mouse Prostate (TRAMP) model reduced the incidence of poorly differentiated prostatic adenocarcinoma by 7.7 fold. This decrease was accompanied by more than 50% decrease in prostate cell proliferation, IGF-1 levels and its downstream effectors- phosphor ERKs 1 & 2 in the prostate 78. The inhibition of carcinogenic pathways was noted in a study showing that administration of 1 gram of resveratrol daily for 4 weeks significantly inhibited plasma cytochrome p450 enzymes such as 2D6, 2C9, CYP1A2 and CYP3A4 compared to baseline 85. Resveratrol also promotes immune-surveillance by modulating natural killer (NK) cells by eliminating spontaneous tumor cells. Clinical studies showed a correlation between administration of 1 gram resveratrol per day and increased expression of NKG2D antigen receptors on NK cells in the blood 86. Recently, it was shown that resveratrol acts as an epigenetic regulator owing to its ability to alter DNA methylation and miRNA expression 87, 88. Its role in inhibiting colorectal cell invasion as well as in attenuating angiogenic responses and suppressing tumor metastasis has also been reported 89, 90.

### Quercetin

Quercetin belongs to the class of polyphenolic flavonoid compounds ubiquitous in plant food sources and its average daily intake has been estimated at 25mg 91. Animal and human studies have demonstrated that it is absorbed very well with an elimination half life of 25 hrs 91. Serum concentrations of quercetin required for anti-cancer activity seems to be greater than 10μM 92**.** A single dose of up to 4 gm was not associated with any side effects 92**.** It has been shown to possess mutagenic activity in the Ames test 93 but not carcinogenic activity 94. Major molecular mechanisms of action of quercetin include down regulation of mutant p53 in breast cancer cells leading to G1 phase arrest of cell cycle 95, tyrosine kinase inhibition both *in vitro* and *in vivo* human studies 96, inhibition of heat shock protein and Ras proto-oncogene 97. A number of these actions have also been demonstrated in ovarian cancer cells. It inhibited heat shock protein-70 98. It has been shown to sensitize with cisplatin in inhibiting SK-OV-3 and CAOV3 ovarian cancer cell proliferation 99. Further Scambia *et al*. 100, 101 showed that quercetin treatment of OVCAR 433 ovarian cancer cells increased TGF-β secretion over 24 hrs and this could be the cause for inhibition of cell proliferation, as administration of a monoclonal antibody to TGF-β reversed these effects of quercetin. In cervical cancer, quercetin showed to reduce cell viability, colony formation and enhanced G_2_-M cell cycle arrest, DNA damage and apoptosis 102. Furthermore, quercetin showed to inhibit PI3K, MAPK and WNT pathways 102. Similarly, in melanoma cells, quercetin significantly reduced cellular viability and proliferation, and induced apoptosis involving JNK/P38 MAPK signaling activation 103.

### Dihydroartemisinin (DHA)

DHA is a derivative of artemisinin, a compound extracted from the wormwood *Artemisia annua*, a species belonging to the daisy family and used by ancient Chinese herbalists to treat fevers. It has been shown to kill a variety of cancer cells by inducing apoptosis. It is cytotoxic to papilloma virus-expressing epithelial cells *in vitro* and *in vivo* 104 and induced apoptosis through activation of mitochondrial caspase pathway in a p53 independent manner. It downregulates VEGF expression in RPMI18226 multiple myeloma cells 105. and further inhibits proliferation, migration and tube formation of HUVE cells. It exerts cytotoxic effects on C6 glioma cells and inhibits hypoxia inducible factor-1 α activation 106. DHA has been shown to synergize with temozolomide to exert cytotoxic effects in rat C6 glioma cells 107. It has been shown to bind to human fortillin; (an anti-apoptotic molecule overexpressed in many cancers) increased its ubiquitination and degradation. It induced DNA fragmentation in U205 cells in a fortillin dependent manner. Data indicate that fortillin is a molecular target of DHA. Developed as an antimalarial drug, it has undergone *in vivo* testing in animals and humans. It has shown to sensitize human ovarian cancer cells to carboplatin therapy 108. Jiao *et al* 109 demonstrated that a panel of ovarian cancer cells were susceptible to treatment with dihydroartemisinin alone; the cancer cells lines were 5-10 fold more sensitive as compared to normal ovarian cell lines. It induced G2 cell cycle arrest, decrease in anti apoptotic proteins Bcl-xL, Bcl-2, increase in pro apoptotic proteins Bax and Bad 109.

### Procyanidin

Procyanidin is found at high concentration in cocoa, berries, apple and grapes. Procyanidin increases mRNA expression of the tumor suppressor gene IGF-2R and PTEN, and is also, a potent inhibitor of P-gp (a multidrug resistant gene) 33, suggested additive to supplement conventional therapy. Recent evidence supports the chemo-preventive potential of procyanidin in lung and breast cancer 33, 110, 111.

### Folate and folic acid

Folate and Folic acid are extracted from asparagus, green leafy vegetables and broccoli 112. Pre-clinical studies showed folate is involved in DNA repair and modulation of S-adenosylmethionine; a donor of a methyl group for DNA methylation. Multiple large-scale studies have suggested that dietary folate lowers risk of various cancers including stomach, lung, pancreatic, colorectal and breast cancer 113, 114.

### Lycopene

Lycopene, a carotenoid extracted from vegetables and fruits is found in high concentration in tomatoes 115. Lycopene reduces intercellular reactive oxygen species (ROS) by promoting antioxidants such as glutathione-S-transferase-omega-1 and superoxide dismutase-1 (SOD-1); by promoting ERO-1. Evidence shows that lycopene reduces ovarian tumor growth 116, leads to a reduced risk in breast and prostate tumor, and can also significantly inhibit the cellular growth of colorectal 117 and lung cancer 118. Importantly, lycopene can also alleviate radiation-induced esophagitis 119 and cisplatin-induced nephropathy 120.

### Sulforaphane (SFN)

Sulforaphane (SFN) is found in high concentrations in cauliflower, broccoli, and cruciferous vegetables. SFN was shown to suppress tumor angiogenesis by reducing cell viability, migration and tube formation in HUVEC-epithelial cell lines possibly through inhibition of signalling between STAT3/HIF-1α/VEGF 118. It was recently shown to reduce YAP1 signaling leading to a reduction in cancer stem cell survival and tumor formation in epidermal squamous cell carcinoma 121.

### Indole-3-Carboinol (I3C)

Indole-3-carbinol (I3C), a phytochemical extracted from cruciferous plants such as broccoli, cabbage and cauliflower, is known to prevent cancer development 122. Its ability to cause G1 arrest of cell cycle, induction of apoptosis and to interfere with signal transduction pathways have been demonstrated in a variety of cancer cell lines, including prostate 123, melanoma 124 and breast cancer 125. I3C protects the cell from oxidative stress resulting from the formation of reactive oxygen species (ROS). In the breast cancer cell line, MDA-MB-231, 3'-diindolylmethane, another member of I3C, suppresses activity of the Akt/NF-кB signaling pathway, promoting apoptosis and inhibits angiogenesis 122. Ability of this compound to target multiple genes involved in both cell cycle (cyclin D1, cyclin E, cyclin-dependent kinases CDK2, CDK4 and CDK6) and apoptosis (Bcl-2, Bcl-xl, survivin, inhibitor of apoptosis protein IAP, x-chromosome linked IAP, pro-apoptotic gene Bax, activation of caspase-9 and caspase 3) have been well documented 126. In addition, it has been shown to inhibit activation of transcription factors including nuclear factor-kappa B, SP1, estrogen receptor, androgen receptor and nuclear factor-E2-related factor 2 (Nrf2). It has a strong hepatoprotective activity against various carcinogens. Due to its broad spectrum of activities, combined with low toxicity, I3C has been acclaimed as a potent chemopreventive and anti-cancer agent 127. The effects of this compound on various cancers and mechanisms of action have been reviewed 126, 128. Howells *et al*. 43 have reviewed the bioavailability of I3C, and showed that plasma levels of 4 µg/ml was achieved with feeding a dose of 250 mg/kg. Work in our laboratory demonstrated a synergistic effect of resveratrol with I3C to inhibit ovarian cancer cell proliferation 122, 129. The effects of resveratrol on cell proliferation and G2/M phase of cell cycle were potentiated by addition of I3C 122, 129.

### Ellagic acid

Ellagic acid, found in *Punica granatum* (pomegranate), exhibits anti-proliferative capacities and promotes apoptosis in prostate cancer cells via the cyclin kinase inhibitor-cyclin cdk machinery. Ellagic acid down regulates Bcl-X_L_ and BcL-2; cyclins D1, D2, cdk 2, 4 and 6 130. Ellagic acid was also shown to reduce metastasis in breast 131 and ovarian cancer 132. Studies have indicated ellagic acid to have anti-cancer effects due to its ability to target histone deacetylase 6-related pathways 133.

### Genistein

Genistein**,** an isoflavone found primarily in soybeans, modulates multi-signalling targets in cancer cells. This bioactive compound modulates the cell cycle and apoptosis process of prostate, breast and lung cancer cell lines by inhibiting NF-kB and Akt signalling pathway and promotes G2/M and G0/G1 arrest in various cancer cell lines 134. Genistein downregulates anti-apoptotic proteins BcL-X_L_ and BcL-2 and upregulates pro-apoptotic Bax, Bak, and Bad proteins 134. Chemotherapeutic agents such as docetaxel, gemcitabine and cisplatin promote NF-kB activation in cancer cells - a possible cause of drug resistance 135. Pre-treatment with genistein in *in-vivo* and *in-vitro* models reduced cell growth and promoted apoptosis when compared to chemotherapeutic treatments alone 136.

### Diallyl disulfide

Diallyl disulfide found in cauliflower, broccoli and cabbage was shown to promote G2/M cell cycle arrest by generating ROS and reducing the HDAC activity in cancer cells 137.

## Perspectives: Phytochemical Combination Studies

Many of these herbs and their extracts are further mixed and prescribed in Asian countries for chronic diseases such as arthritis, diabetes and cancers. There have been many claims by the practitioners in India, China and other asian countries, of having cured cancers. The senior author has met several of them, visited their 'clinics', seen their 'patients' who have been claimed to have been rescued from terminal stage neuroblastoma and adenocarcinomas. A common feature of these practitioners has been a reluctance to subject their herbal preparations to clinical trials using accepted parameters (double blind, randomized, cross-over studies or case control studies). In the US, they are not approved by the regulating bodies such as FDA for treating specific diseases, but are sold extensively over the counter as nutritional dietary supplements. Research in this exciting area has been intense and has succeeded in identifying several active compounds (such as the ones described in this review), purification, characterization and structure elucidation of these compounds and structure-function studies leading to identification of more potent analogues. Molecular mechanisms of action of these compounds is being investigated widely 122, 129. Many of these have been tested individually and in combination of 2 or 3 together and have shown additive and synergistic properties 122, 129. However, combination studies using several of these together have not been explored, and molecular mechanisms of such combinations need to be investigated. The ability of such multiple combinations to cause complete and permanent remission of cancers need to be evaluated.

The preceding discussion of literature on these various phytochemicals demonstrates that 1) each of these chemicals has multiple effects and targets multiple pathways in the cancer cell, 2) an additive or synergistic action was noted upon combination of two and sometimes three compounds 122, 129, 3) a striking feature of low toxicity combined with effective absorption upon oral administration , 4) many of these compounds have effects on, not one but many different cancers, and 5) each of these pyto-chemicals has many common mechanisms with others, and acts on pathways in a manner unique to themselves. Molecular mechanism of action of these compounds is being investigated widely 122, 129. Many of these have been tested individually and in combination of two and have shown additive and synergistic properties. However, combination studies using several of these together have not been investigated and molecular mechanisms of such combinations need to be determined. The ability of such multiple combinations to cause complete and permanent remission of cancers need to be evaluated.

Our recent work demonstrated synergism between several compounds 122, 129. Our recent results demonstrated the effectiveness of different combinations in causing 100% inhibition of breast cancer cell proliferation, by a combination of five phytochemicals used at bioavailable concentrations (122, 129. These results pave the way to evaluate their effectiveness *in vivo*. The main objective of our proposal is to elucidate unique molecular mechanisms that contribute to increased effectiveness of these phytochemicals when used in combination to inhibit ovarian epithelial carcinoma. This data prompted us to generate several hypotheses that: 1) Cell proliferation inhibitory activity of phytochemical combination is the result of synergistic and additive actions of the individual selected phytochemicals; 2) combination of these phytochemicals activates gene(s) that are exclusive to the combination; 3) identification of these gene(s) unique to the combination, have the potential to be used for the design of targeted therapy of BC and 4) this combination of five will be effective in chemoprevention and chemotherapy of various cancers in *in vivo*. Our ongoing *in vivo* studies will shed light on the possibility of establishing specific phytochemical combinations to treat specific cancers. More interestingly, our genomic studies, using microarray or RNAseq technologies combined with functional studies will allow us the identification and validation of candidate genes that will pave the way towards the design of effective targeted therapeutic cancer strategies.

## Conclusion

Cancer is a complex disease with various aetiologies, and conventional therapies cause multiple side effects, representing a serious economic challenge. There are more than 5000 phytochemicals found in fruits, grains and vegetables with a large amount still undiscovered 138. The efficacy of a combination of phytochemicals in cancer therapy, maybe due to their ability in modulating different signaling pathways, at once, that promote cell death, inhibit cell proliferation and invasion, sensitize cancerous cells, and boost the immune system. Combinations of cytotoxic anti-tumour agents and inhibitors from phytochemicals might act together producing inhibitory mechanisms against tumor growth. While the combination of conventional and CAM therapies is a promising strategy, clinical studies are required to establish better anti-cancer treatments 139. Moreover, preclinical and epidemiological studies are required to identify the molecular signatures of newly discovered phytochemicals and identify their associated carcinogenesis biomarkers.

## Figures and Tables

**Figure 1:**
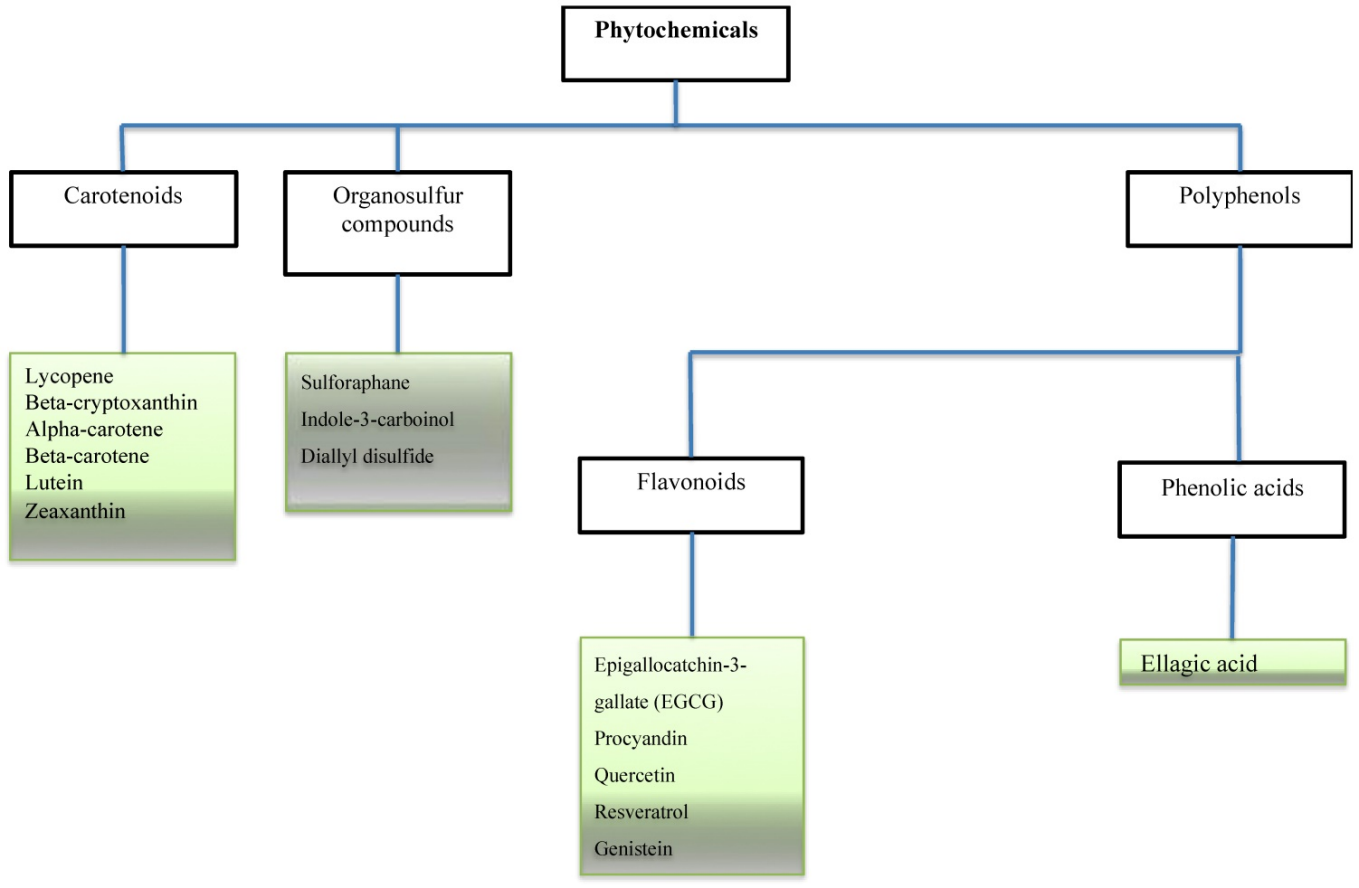
** Phytochemicals in cancer related studies.** The Figure illustrates the most studied phytochemicals that can inhibit tumor growth and progression.

**Figure 2:**
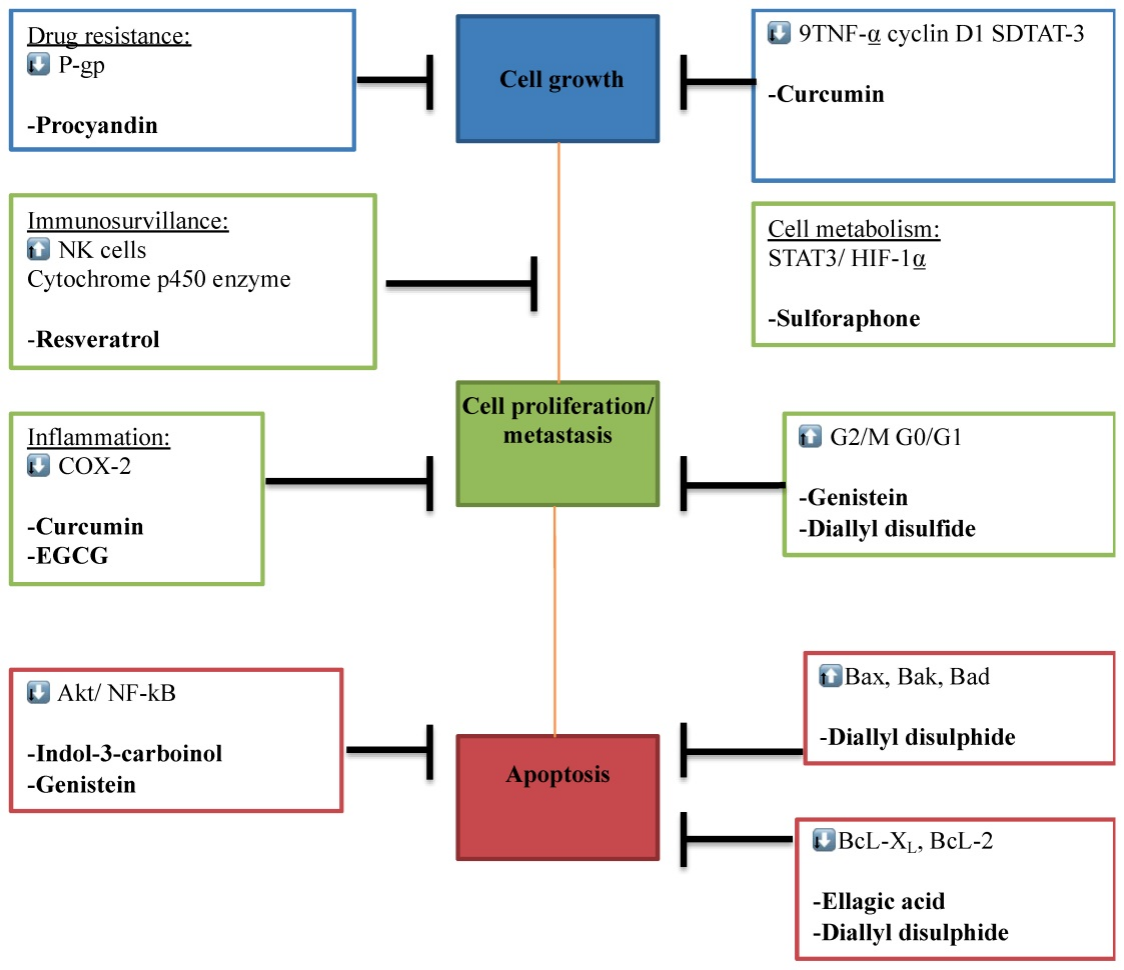
** Multi-molecular anticancer target of phytochemicals.** The Figure illustrates the different phytochemicals and their corresponding molecular targets that underpin their effects on tumor cell proliferation, invasion and metastasis, immune surveillance, drug resistance, inflammation, and tumor cell metabolism.

**Table 1:** The different phytochemicals and their corresponding molecular targets underpinning effects on tumor growth, differentiation, proliferation, invasion and metastasis, drug resistance, immune surveillance, inflammation and tumor cell metastasis *via* different pathways.

Source	Active Ingredient	Literature
**Broccoli**	**-Indole-3-Carbinol (I3C)**	I3C induces apoptosis, antiangiogenic activities and growth inhibition in multiples cancer cell lines and tumours 124, 140. I3C induces G1 cell cycle arrest 141. I3C inihbits Akt 142. I3C inhibits activation of transcription factors including nuclear factor-kappa B, SP1, estrogen receptor, androgen receptor and nuclear factor-E2-related factor 2 (Nrf2) 127. I3C has broad spectrum of activities, combined with low toxicity 137. I3C up-regulated the tumor suppressor protein p23 and down-regulated cell cycle check protein pRb and survival indicator Survivin 122, 129.
**Grape skin and seeds**	**Resveratrol (RE)**	RE interferes with AKT activity and triggers apoptosis 143. RE enhances p53 acetylation and apoptosis 144. RE causes G1 arrest 145. RE induces apoptosis by TRAIL sensitization 146 and down regulates survivin expression 122. Resveratrol blocks proliferation, migration and invasion via NEAT1-mediated Wnt/β-catenin signaling pathway 147.
**Tea**	**Epigallo-Catechin Gallate (ECG)**	ECG inhibits and Hsp70 and Hsp90 functions 148, 149. ECG inhibits hypoxia and serum induced HIF-1 alpha protein accumulation and VEGF expression 150, 151. Combination studies have shown that ECG can enhance responses induced by curcumin on breast cancer cells 152.
**Spirulina**	**Phycocyanin (P)**	P inhibits cell proliferation and induces apoptosis due to the MAPK, Akt/mTOR/p70S6K and NF-κB pathways 153. P inhibits MDR1 through reactive oxygen species and cyclooxygenase-2 mediated pathways 154. Potent dietary phyto-antioxidant, anti-inflammatory and anti-cancerous 82
**Turmeric roots**	**Curcumin (CUR)**	CUR has anti-proliferative effect 83 and reduces EMT 48. CUR induces apoptosis by blocing the PI3K/AKT Pathway 155. CUR down regulates cyclin D1, cyclin E, MDM2 and up regulates tumor suppressors p21, p27 and p53 156, 157.
**Stamens of Saffron**	**Crocin (Cr)**	Saffron inhibits DMBA-induced skin carcinoma 158. Saffron causes cell death through apoptosis 159. Animal and *in vitro* studies indicate that saffron and its main constituents such as crocusatin H, crocin-1 and crocin-3 possess anticancer and anti-tumour activities 160.
